# Plane xanthoma

**DOI:** 10.1016/j.jdcr.2025.04.027

**Published:** 2025-06-25

**Authors:** Connor A. Sheehan, Theresa N. Tran, Jason B. Lee, Jeffrey Y. Liu, Sylvia Hsu

**Affiliations:** a Department of Dermatology, Temple University Lewis Katz School of Medicine, Philadelphia, Pennsylvania; b Department of Dermatology and Cutaneous Biology, Sidney Kimmel Medical College at Thomas Jefferson University, Philadelphia, Pennsylvania

**Keywords:** plane xanthoma, primary biliary cholangitis

## Case presentation

**Fig 1:**
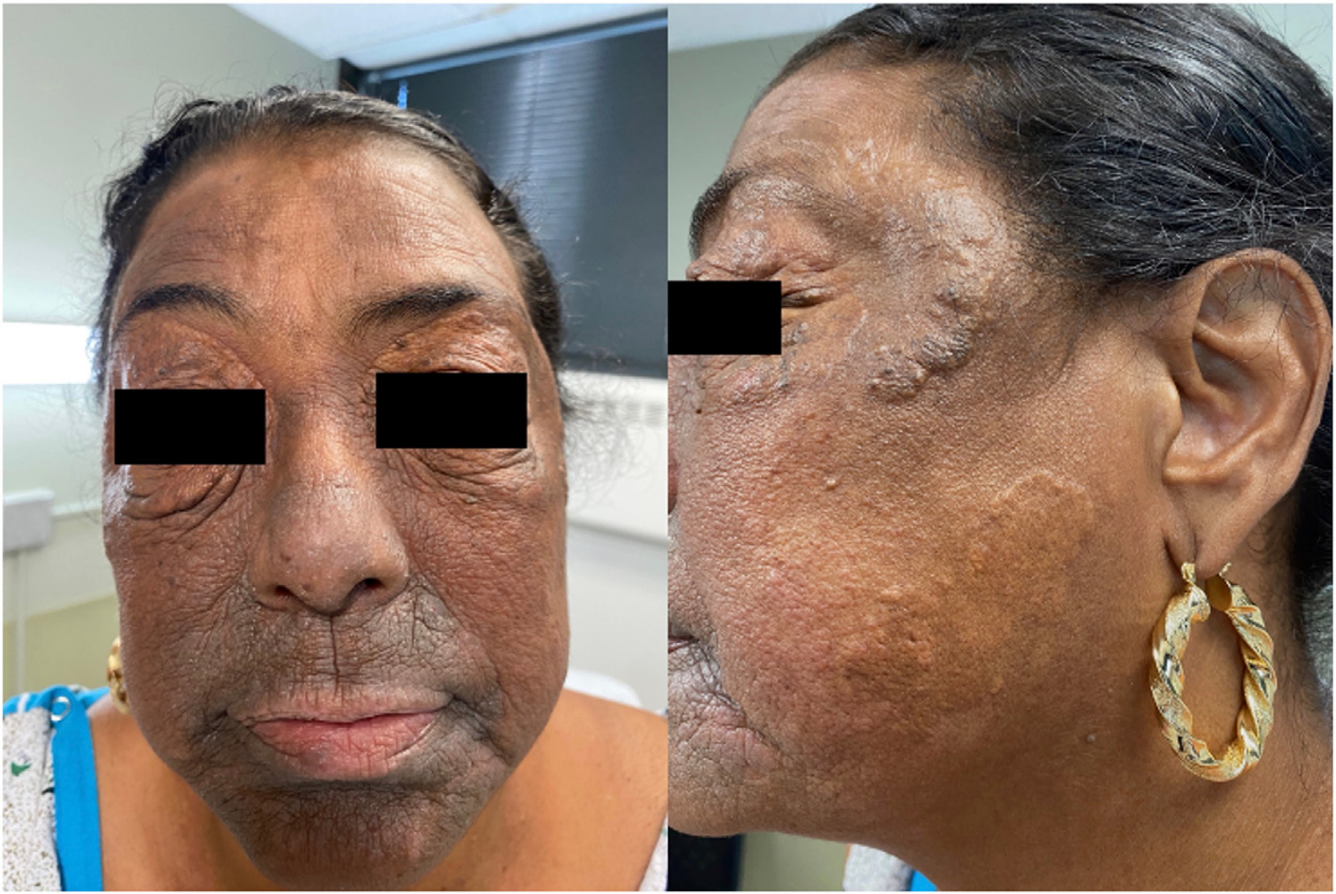
Yellow plaques on the face.

**Fig 2:**
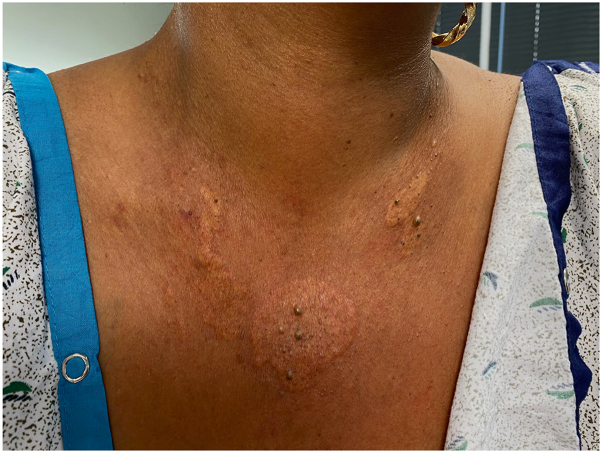
Yellow plaques on the chest.

**Fig 3:**
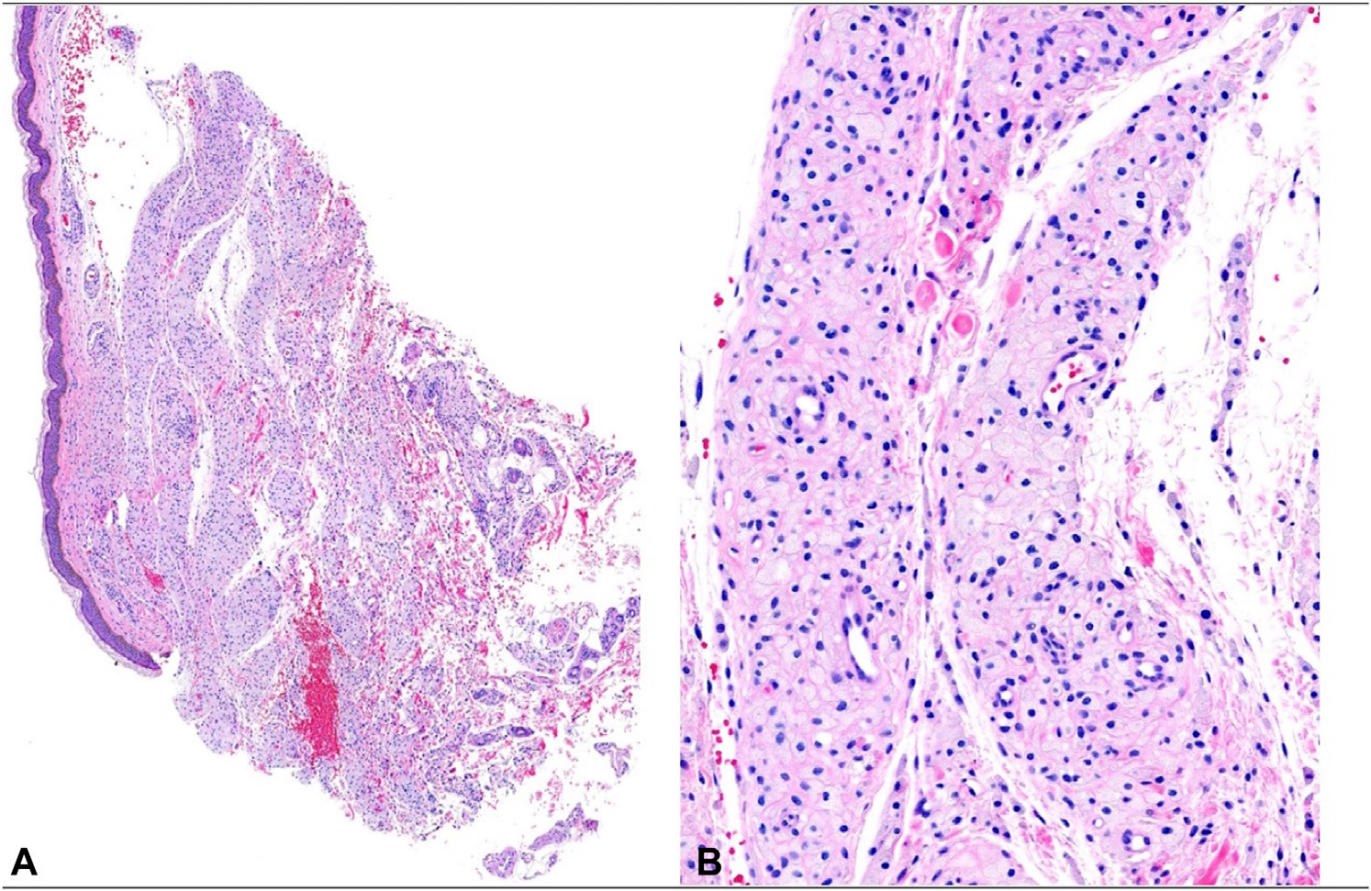
**A,** Histopathology showing pale cells throughout the dermis (H&E 40×). **B,** Histopathology showing a diffuse collection of pale cells throughout the dermis and many lipid-laden foamy histiocytes (H&E 200×). *H&E*, Hematoxylin and eosin.

A 46-year-old woman was referred from her hepatologist for an eruption on her face and chest that had been present for several years. Three years earlier, she had been referred to the hepatologist by her primary care physician due to abnormal liver function tests. Laboratory evaluation showed a cholestatic and hepatocellular enzyme elevation pattern, with markedly elevated alkaline phosphatase, aspartate aminotransferase, and alanine aminotransferase. Autoimmune serologies were notable for a high-titer positive antinuclear antibodies (1:1280) and positive antismooth muscle (1:80) and antimitochondrial (1:20) antibodies. The lipid panel revealed significant hypercholesterolemia. The patient had undergone a liver biopsy and was diagnosed with autoimmune hepatitis (AIH) and primary biliary cholangitis (PBC) overlap syndrome. Despite adequate medical management, her skin lesions persisted. When she presented to our dermatology clinic, there were well-demarcated, yellow plaques at the periorbital area and lateral mandibular area (Fig 1) and on her chest (Fig 2). Two 4-mm skin punch biopsies of her neck and chest showed a diffuse collection of pale cells throughout the dermis and many lipid-laden foamy histiocytes, confirming our clinical diagnosis of plane xanthoma (Fig 3, *A* and *B*).

**Question: What is the most likely etiology for this patient’s plane xanthomas?**

**A.** AIH and PBC overlap syndrome
**B.** Familial hypercholesteremia
**C.** Familial hypertriglyceridemia
**D.** Multiple myeloma
**E.** Systemic lupus erythematosus

**Answer Discussion**

The correct answer is (A) AIH and PBC overlap syndrome. The formation of xanthomas due to secondary hyperlipidemia in conditions such as PBC has been extensively described, with xanthomas reported to occur in almost 6% of patients with PBC.^1^ Plane xanthomas in association with PBC often begin on the hands and feet and can become generalized.^2^^,^^3^ It is hypothesized that the formation of plane xanthomas of cholestasis is due to the accumulation of unesterified cholesterol, specifically lipoprotein X, leading to significant dyslipidemia and skin findings.^4^ Resolution of plane xanthomas typically requires treatment of the underlying cholestasis. Medical management of dyslipidemia is first-line but rarely resolves xanthomas; thus, liver transplantation may be necessary for restoring biliary drainage, lipid-level stability, and subsequent xanthoma improvement.^5^ In addition to surgical excision, cosmetic therapies such as chemical peels or lasers are temporizing measures. They do not address the underlying cholestasis, and the lesions may reoccur.^5^ Plane xanthomas in normolipemic patients are less common, but may indicate an underlying monoclonal gammopathy (often a plasma cell dyscrasia) or a lymphoproliferative disorder (B cell lymphoma, Castleman disease), as these conditions may disrupt the normal metabolism of lipoproteins via binding between circulating immune complexes and plasma lipids leading to phagocytosis by macrophages and deposition in the skin.^2^^,^^4^ The location of plane xanthomas is sometimes pathognomonic for specific etiologies. For example, intertriginous plane xanthomas are associated with homozygous familial hypercholesterolemia and palmar crease plane xanthomas coexisting with tuberoeruptive xanthomas are found in patients with familial dysbetalipoproteinemia type III.^4^

## Conflicts of interest

None disclosed.
